# Overlooked gall-inducing moths revisited, with the description of *Andescecidiumparrai* gen. et sp. n. and *Olierasaizi* sp. n. from Chile (Lepidoptera, Cecidosidae)

**DOI:** 10.3897/zookeys.795.27070

**Published:** 2018-11-12

**Authors:** Gabriela T. Silva, Gilson R.P. Moreira, Héctor A. argas, Gislene L. Gonçalves, Marina D. Mainardi, Germán San las, Donald Davis

**Affiliations:** 1 PPG Biologia Animal, Departamento de Zoologia, Instituto de Biociências, Universidade Federal do Rio Grande do Sul, Av. Bento Gonçalves 9500, Porto Alegre, RS, 91501-970, Brazil; 2 Departamento de Recursos Ambientales, Facultad de Ciencias Agronómicas, Universidad de Tarapacá, Casilla 6-D, Arica, Chile; 3 PPG Genética e Biologia Molecular, Departamento de Genética, Instituto de Biociências, Universidade Federal do Rio Grande do Sul, Av. Bento Gonçalves 9500, Porto Alegre, RS, 91501-970, Brazil; 4 Ciências Biológicas, Instituto de Biociências, Universidade Federal do Rio Grande do Sul, Av. Bento Gonçalves, 9500, Porto Alegre, RS 91501-970, Brazil; 5 Facultad de Ciencias Exactas y Naturales, CONICET, Universidad Nacional de La Pampa, La Pampa 6300, Argentina; 6 Department of Entomology, National Museum of Natural History, Smithsonian Institution, Washington, DC 37012-7012, USA

**Keywords:** Anacardiaceae, Cecidosid moths, insect galls, Neotropical microlepidoptera, *
Schinus polygamus
*

## Abstract

There are still many gall systems associated with larvae of Lepidoptera in which the true gall-inducers have not been identified to species. Reports on misidentification of gall inducers have been recurrent for these galls, particularly in complex gall-systems that may include inquilines, kleptoparasites, and cecidophages, among other feeding guilds such as predators and parasitoid wasps. Here we describe and illustrate the adults, larvae, pupae and galls, based on light and scanning microscopy, of *Andescecidiumparrai***gen. et sp. n.** and *Olierasaizi***sp. n.**, two sympatric cecidosid moths that are associated with *Schinuspolygamus* (Cav.) Cabrera (Anacardiaceae) in central Chile. Adults, immatures, and galls of the former did not conform to any known cecidosid genus. Galls of *A.parrai* are external, spherical, and conspicuous, being known for more than one century. However, their induction has been mistakenly associated with either unidentified Coleoptera (original description) or *Olieraargentinana* Brèthes (recently), a distinct cecidosid species with distribution restricted to the eastern Andes. Galls of *O.saizi* had been undetected, as they are inconspicuous. They occur under the bark within swollen stems, and may occur on the same plant, adjacent to those of *A.parrai*. We also propose a time-calibrated phylogeny using sequences from mitochondrial and nuclear loci, including specimens of the new proposed taxa. Thus in addition to clarifying the taxonomy of the Chilean cecidosid species we also tested their monophyly in comparison to congeneric species and putative specimens of all genera of Neotropical and African cecidosids.

## Introduction

Cecidosidae are a small group of ancient gall-inducing and stem-mining micromoths restricted in distribution to the Southern Hemisphere. They include seven genera and 19 species that are distributed in southern South America, southern Africa, and New Zealand (for taxonomic reviews see Davis 1988, Hoare and Dugdale 2003, Moreira et al. 2017). In spite of their interesting biology, close association with their Anacardiaceae host-plants (*Schinus* L.) and broad geographical distribution, the diversity of Cecidosidae remains mostly unexplored in the Neotropical region. Their galls may consist of complex ecological systems that involve additional organisms, in addition to the host-plant and corresponding gall-inducer. For example, other insects can act in multi-trophic levels in these galls, as inquilines, kleptoparasites, or cecidophages, among other feeding guilds, including predators, parasitoids or successors that use them just for shelter (Moreira et al. 2017). In the case of kleptoparasites and inquilines, the original inducer might be killed and thereafter the gall is usurped by the invader (Ronquist 1994, van Noort et al. 2007). This type of phenomenon may bring important taxonomic consequences, preventing the correct identification of the real inducer when such systems are undersampled. This situation is found for many gall-inducing insects, including other Lepidoptera (e.g., Morris 2000, van Noort et al. 2007, Luz et al. 2015). This was recently demonstrated in detail for *Cecidoniuspampeanus* Moreira and Gonçalves, a cecidosid moth whose identity was overlooked for more than one hundred years, as its galls had been erroneously described as induced by their hymenopteran inquilines (Moreira et al. 2017).

A similar case of such a complex system was recently found for a cecidosid-induced gall in central Chile. In fact, such a spherical, external gall was briefly described for the first time by Kieffer and Herbst (1905), in association with the branches of the Anacardiaceae*Duvauadependens* (Ortega) DC. [= *S.polygamus* (Cav.) Cabrera] as induced by an unidentified species of *Bruchus* L. (Coleoptera, Chrysomelidae). One year later, the same authors reported this type of gall again, providing in addition illustrations that included a cross-section view of it (Kieffer and Herbst 1906). In his monograph of galls from South and Central American plants later, Houard (1933) reproduced this description and illustrations, associated with the same country, gall-inducer, and host-plant. More than 50 years later, Núñez and Sáiz (1994) working with these galls in the Parque Nacional La Campana, Valparaiso Region, cited the corresponding findings that were repeated by Houard (1933); however, a note was added by the authors recognizing that the true inducer of the galls belonged to Lepidoptera. Later, Sáiz and Núñez (1997) stated that these were in fact induced by Cecidosidae, but they did not describe the moth. Based on scarce material sent by these authors to the Universidad de Concepción, Parra (1998) described for the first time the corresponding gall-inducer cecidosid moth as conspecific to *Olieraargentinana* Brèthes, a species at that time only known to occur on the eastern side of the Andes (Argentina) and with poorly known immatures. He also synonymized *Oliera* Brèthes with *Cecidoses* Curtis, and proposed *Cecidosesargentinana* (Brèthes), thereafter considered to be the valid species responsible for the induction of *Schinus* galls in Chile. However, Moreira et al. (2012) in a broad study including abundant material of galls from Argentina and Brazil, also including type material, confirmed the original description of *O.argentinana* given by Brèthes (1916), showing that *Oliera* galls are formed under the bark instead, and have different morphology from material described by Parra (1998) based on the larvae, pupae and adults, thus revalidating the genus *Oliera*. In addition to morphological characters, Moreira et al. (2012) based their study on a preliminary analysis of mitochondrial (COI) DNA sequences, including putative members of the four Neotropical cecidosid genera recognized at that time. They also concluded that material described by Parra (1998) should belong to a different cecidosid genus yet to be identified, which is described in detail in the present study.

While searching later in the field for additional material of this new cecidosid genus, three of the authors of this study (G. R. P. Moreira, G. San Blas and H. A. Vargas) found that there was a second undescribed, cecidosid species in Chile occurring in sympatry, forming galls on swollen stems of the same *S.polygamus* plants, as already suggested by Sáiz and Núñez (1997). These internal, inconspicuous galls, from which Parra (1998) had not received material to work at that time, turned out to be an additional, unknown species of *Oliera* (Brèthes), which is also described here. Thus the main goal of this report is to clarify the peculiar and somewhat confused taxonomic history of these two cecidosid moths in Chile. Under light and scanning electron microscopy, their galls, larvae, pupae and adults are described and illustrated, and preliminary information on their life history is provided. A phylogenetic analysis of concatenated mitochondrial (COI and 16S) and nuclear (Wingless) DNA sequences, including putative members of all lineages of extant Cecidosidae from South America and Africa, was also conducted to provide support for the proposition of these new taxa.

## Materials and methods

### Morphology

Adults used in the study were reared by H.A. Vargas from galls either attached to *Schinuspolygamus* branches or detached ones found in litter, under the canopy cover, which were maintained in small plastic vials at room temperature in the entomology laboratory of the Facultad de Ciencias Agronómicas, Universidad de Tarapacá, Arica, Chile. They were periodically inspected for emerged adults, which were then pinned and dried. Larvae and pupae were obtained by dissecting additional galls. They were fixed in Kahle-Dietrich´s fluid and preserved in 70% EtOH. For DNA analyses, additional specimens were preserved in 100% EtOH at -20 °C.

For gross morphology studies, the specimens were cleared in a 10% potassium hydroxide (KOH) solution and mounted on slides with either glycerin jelly or Canada balsam. Observations were made with the aid of a Leica M125 stereomicroscope. Structures selected to be drawn were previously photographed with an attached Sony Cyber-shot DSC-H10 digital camera. The vectorized line drawings were made with the software CorelPhotoPaint X4, using the corresponding digitalized images as a guide. At least four specimens were used for the descriptions of each life stage. Measurements were made with an attached ocular micrometer; values are presented as mean ± standard deviation unless noted otherwise.

Specimens used in scanning electron microscope (SEM) analyses were dehydrated in a Bal-tec CPD030 critical-point dryer, mounted with double-sided tape on metal stubs and coated with gold in a Bal-tec SCD050 sputter coater. They were examined and photographed in a JEOL JSM6060 scanning electron microscope at Centro de MicroscopiaEletrônica (CME) of Federal University of Rio Grande do Sul, (UFRGS), Porto Alegre, RS, Brazil.

The material examined is deposited in the following collections:

**IADIZA** Instituto Argentino de Investigaciones de las Zonas Áridas, CCT-CONICET Mendoza, Mendoza, Argentina;

**IDEA** Colección Entomológica de la Universidad de Tarapacá, Arica, Chile;

**LMCI** Laboratório de Morfologia e Comportamento de Insetos /UFRGS, Porto Alegre, Brazil;

**MNNC**Museo Nacional de História Natural, Santiago, Chile;

**UNCC**Museo de Zoologia de la Universidad de Concepción, Concepción, Chile;

**USNM**United States National Museum-Smithsonian Institution, Washington DC, USA.

### Molecular phylogeny

We assembled data from two mitochondrial loci and one nuclear protein-coding locus from 12 species representing all Neotropical lineages of Cecidosidae (*Cecidonius* Moreira & Gonçalves, *Cecidoses*, *Dicranoses* Kieffer & Jörgensen, *Eucecidoses* Brèthes and *Oliera*) to infer the relationship of *Andescecidium* gen. n. and the phylogenetic status of *A.parrai* sp. n. and its congeneric undescribed taxon *Andescecidium* sp. (Table 1). To infer the relationship of *O.saizi* sp. n., we added an undescribed taxon (*Oliera* sp.) in addition to the type species (*O.argentinana*). We also included distinct populations of *A.parrai* and *O.saizi* to cover putative geographic divergence. The South African genus *Scyrotis* Meyrick was also taken into account in the analysis. As outgroup, we included representative species of Adelidae, Incurvariidae, Heliozelidae, and Prodoxidae, which together with Cecidosidae comprise the superfamily Adeloidea (van Nieukerken et al. 2011, Regier et al. 2015). Voucher specimens used are listed in Table 1.

Total genomic DNA was purified from fresh larval tissue of *Andescecidiumparrai*, *Andescecidium* sp. and two additional species of *Oliera* (*O.saizi* and *Oliera* sp.), using the PureLink genomic DNA kit (Life, Invitrogen, USA) following the manufacturer’s instructions. Sequences of *Cecidoniuspampeanus*, *Cecidonius* sp., *Cecidoseseremita* Curtis, *Dicranosescongregatella* (Brèthes), *Eucecidosesminutanus* Brèthes, *Olieraargentinana*, *Scyrotisgranosa* (Meyrick) were previously generated by Moreira et al. (2017). Adelidae, Incurvariidae and Prodoxidae sequences were incorporated from the GenBank/NCBI databases, as well as the African cecidosid *S.pulleni* Mey.

Approximately 2.5 Kb of gene segments were amplified by polymerase chain reaction (PCR): 1421 bp of the cytochrome oxidase subunit I (COI), 474 bp of the 16S ribosomal RNA (16S) and 395 bp of the Wingless (Wg) gene loci, with primers and conditions described in Moreira et al. (2017). PCR products were purified using the enzymatic method (exonuclease and alkaline phosphatase), sequenced with BigDye chemistry, and analyzed in an ABI3730XL (Applied Biosystems Inc.). Chromatograms obtained from the automatic sequencer were read and sequences were assembled using the software CodonCode Aligner (CodonCode Corporation). Sequences generated in this study will be deposited in GenBank and BOLD (Table 1).

**Table 1.** Specimens used in this study for the phylogenetic inference in Cecidosidae.

Family	Genus	Species	Vouchers*	Pop (#) Locality	GenBank (accession number)			BOLD Systems
					COI	16S	Wg	COI-5P
Cecidosidae	* Andescecidium *	* parrai *	LMCI 231-5	Til-Til/Rungue/Chile	MH750899	–	MH750908	MISA020-18
			LMCI 233-5	Cuesta Barriga/Chile	MH750900	–	MH750906	MISA021-18
			LMCI232-6	Cuesta La Dormida/Chile	MH750901	–	MH750907	MISA022-18
		sp.	LMCI 163-15	Mendoza/Argentina	MH750902	–	–	MISA023-18
	* Cecidonius *	* pampeanus *	LMCI 16-24	Eldorado do Sul/Brazil	MH750864	MH750886	MH750892	MISA024-18
		sp.	LMCI 14-72	Curitiba/Brazil	MH750881	MH750887	MH750893	MISA025-18
	* Cecidoses *	* eremita *	LMCI 163-1	Mendoza/Argentina	MH750879	MH750888	MH750895	MISA026-18
	* Dicranoses *	* congregatella *	LMCI 3-1	Canguçu/Brazil	MH750880	MH750889	MH750896	MISA027-18
	* Eucecidoses *	* minutanus *	LMCI 163-21	Mendoza/Argentina	MH750881	MH750890	MH750897	MISA028-18
	* Oliera *	* argentinana *	LMCI 6-11	Canguçu/Brazil	MH750883	MH750891	MH750898	MISA029-18
		* saizi *	LMCI 232-2	Til-Til/Rungue/Chile	MH750904	–	MH750910	MISA030-18
			LMCI232-4	Cuesta La Dormida/Chile	MH750905	–	MH750909	MISA031-18
		sp.	LMCI 163-13	Mendoza/Argentina	MH750903	–	–	MISA032-18
	* Scyrotis *	* granosa *	LMCI 228-2	Tsitsikamma/South Africa	MH750885	–	–	MISA033-18
		* pulleni *	USNM 00907535	Transvaal /South Africa	-	–	–	LNAUT084-14
Adelidae	* Adela *	* septentrionella *	–	–	EU884115	–	–	–
Incurvariidae	* Incurvaria *	* masculella *	–	–	AF150926	–	–	–
Heliozelidae	* Antispila *	* ampelopsia *	RMNH.INS.30326	–	MF118352	–	–	HELA119-15
Prodoxidae	* Greya *	*enchrisa*	–	–	EU884123	–	–	–

We used BEAST v2.02 (Bouckaert et al. 2014) to perform relaxed clock Bayesian analyses of the concatenated data and of each gene. Input files contained partitions for each codon position of each gene, which were selected by PartitionFinder v.1.1.1 (Lanfear et al. 2012). We linked site models in accordance with partitions identified by PartitionFinder, unlinked clock models across all partitions, and linked topology across all partitions. We tested the null hypothesis of a strict molecular clock for each gene (data not partitioned) using likelihood-ratio tests in MEGA v7 (Tamura et al. 2013) and found that all genes rejected the strict molecular clock hypothesis. Thus we used uncorrelated lognormal relaxed molecular clock models for all partitions, with mean rates estimated from a gamma distribution relative to a partition with an arbitrary fixed rate of 1. For a tree prior, we used a Calibrated Yule (pure-birth) model with random starting trees. Initial runs with BEAST showed that cecidosids did not remain monophyletic in relation to the outgroup taxa (Prodoxidae, Adelidae, Heliozelidae, and Incurvariidae). Because the monophyly of Cecidosidae is not in question in this study, we constrained it to be monophyletic in the analyses. To adjust the molecular clock we used the fossil calibration point of Adeloidea, about 120±10mya, with a log-normal distribution (Walhberg et al. 2013).We used 1×10^5^ generations (sampled every 5,000) for each individual gene tree, with the number of generations increased to 2.5×10^5^ for the concatenated analysis. We assessed convergence of BEAST analyses using Tracer v1.6 (Drummond and Rambaut 2007) to examine whether effective sample sizes exceed 200. We used TreeAnnotator v1.7.5 (Drummond et al. 2006) to summarize the posterior distribution of trees from individual MCMC analyses into maximum clade credibility trees using a burn-in fraction of 25%. Trees were observed and edited in FigTree v1.4.3 (Rambaut 2009). Clades with Bayesian Posterior Probability (BPP) ≥ 95% were considered strongly supported. Pairwise genetic distances (p-distances) among lineages were calculated in MEGA v7 with 1,000 bootstrap replications.

## Results

### Molecular phylogeny

**Figure 1.**
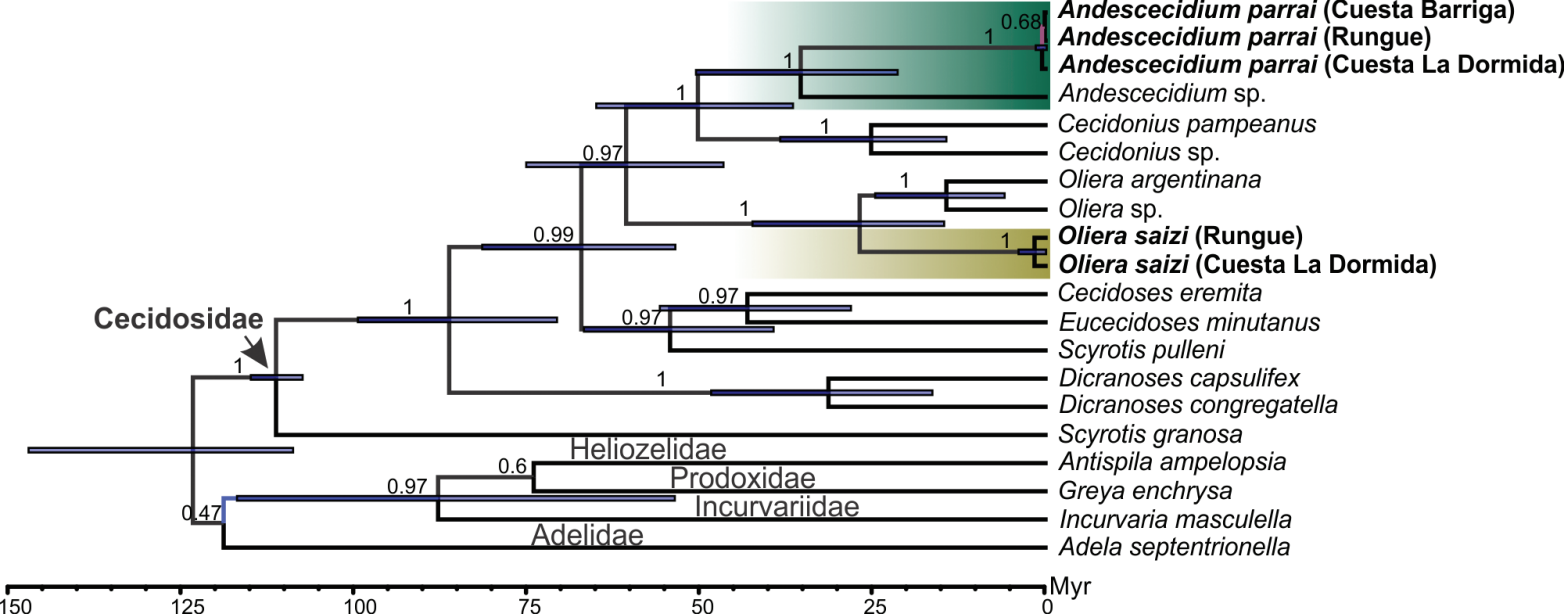
Molecular phylogeny of Cecidosidae showing monophyletic status and relationships of *Andescecidium* and *Olierasaizi* (highlighted by green and brown squares, respectively). Bayesian time-calibrated consensus tree based on cytochrome oxidase subunit I (COI), r16S ribosomal (16S), and Wingless (Wg) genes. Representative species of other Adeloidea families (Adelidae, Incurvariidae, Heliozelidae and Prodoxidae) were used to root the tree. Numbers above branches indicate posterior probability support for the equivalent node. Blue bars indicate confidence interval (95% HDP) for each node age estimate, presented in millions of years ago (Mya).

The concatenated tree corroborated our hypothesis of monophyletic status for the new genus and species and their relationships within Cecidosidae with strong node support (BPP = 1) (Figure 1). Individual trees based on COI and 16S showed nearly identical topologies, with a slight difference for Wg (likely due to evolving at a slower rate than the other genes), resulting in weaker phylogenetic resolution than the concatenated tree; however, all retrieved the monophyletic status of the new taxa (data not shown). The concatenated time-tree indicates that *Andescecidium* is sister of *Cecidonius*, a Pampean lineage found in the grasslands of Southern Brazil, and emerged around 35 Mya (95% HDP 20.6–49.1). Genetic distances of the new genus to other cecidosids ranged from 12 to 27%; a lower divergence was observed in relation to *Olierasaizi* and highest to *Dicranoses* (both *D.congregatella* and *D.capsulifex*) and *Scyrotis* (both *S.pulleni* and *S.granosa* (Table 2).

**Table 2.** Estimates of pairwise genetic distance (%) among Cecidosidae lineages based on concatenated mitochondrial DNA sequences using p-distance.

		1.	2.	3.	4.	5.	6.	7.	8.	9.	10.	11.	12.	13.	14.	15.	16.
1.	* Andescecidium parrai *																
2.	*Andescecidium* sp.	9															
3.	* Cecidonius pampeanus *	12	12														
4.	*Cecidonius* sp.	14	15	7													
5.	* Cecidoses eremita *	16	15	14	15												
6.	* Dicranoses capsulifex *	19	18	19	21	21											
7.	* Dicranoses congregatella *	20	19	20	22	21	9										
8.	* Eucecidoses minutanus *	15	13	13	15	13	21	20									
9.	* Oliera argentinana *	11	13	10	11	12	19	18	11								
10.	* Oliera saizi *	11	12	11	12	12	18	18	13	5							
11.	*Oliera* sp.	12	11	12	15	13	19	18	12	4	6						
12.	* Scyrotis granosa *	21	20	20	23	22	22	23	23	21	20	19					
13.	* Scyrotis pulleni *	16	17	19	19	17	22	21	18	18	16	16	21				
14.	* Adela septentrionella *	20	19	19	21	21	21	21	21	19	16	16	18	21			
15.	* Incurvaria masculella *	20	20	19	22	20	23	24	20	18	19	19	21	22	17		
16.	* Antispila ampelopsia *	23	23	23	25	24	25	25	25	22	22	22	22	25	19	20	
17.	* Greya enchrysa *	19	18	18	21	21	23	24	21	18	20	20	19	21	18	14	18

### Taxonomy

#### Andescecidium

Taxon classificationAnimaliaLepidopteraCecidosidae

Moreira & Vargas gen. n.

http://zoobank.org/5B581601-2C9E-42F7-9E89-1368F6EF38CC

**Figure 2.**
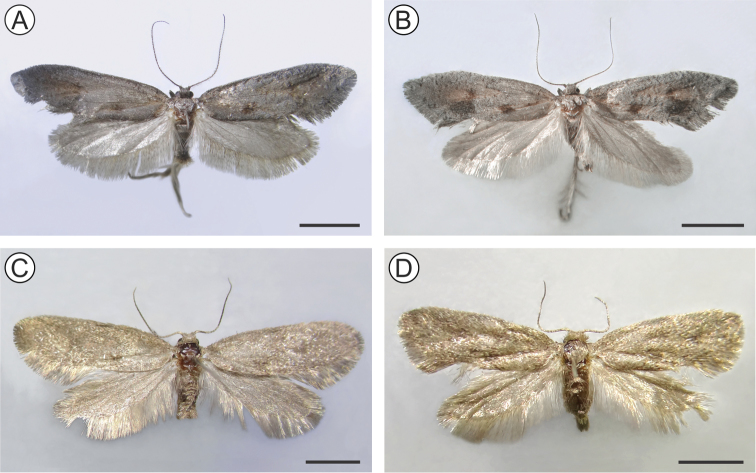
Pinned-dried males (holotypes, left) and females (paratypes, right) of *Andescecidiumparrai* (**A, B**) and *Olierasaizi* (**C, D**), dorsal view. Scale bars: 2 mm (**A, B)**, 1 mm (**C, D**).

**Figure 3.**
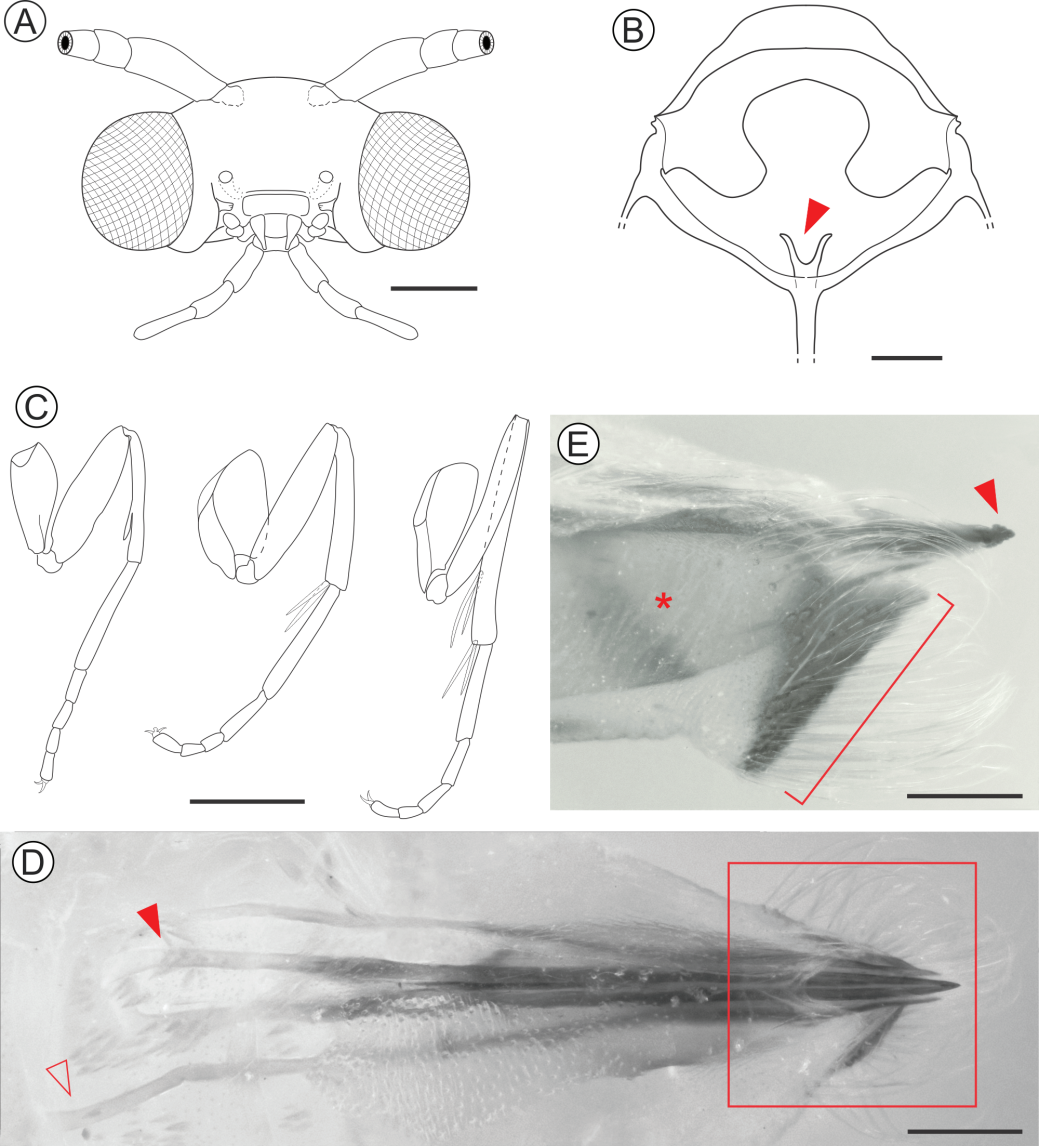
*Andescecidiumparrai* adult morphology under light microscopy (LMCI 218-03). **A** head, anterior view **B** metathoracic furcasternum, posterior (closed arrow points to left furcal apophysis) **C** fore-, median- and hindlegs, from left to right, respectively **D** female genitalia, dorsal (open and closed arrows point to the anterior ends of left anterior and posterior right apophyses, respectively) **E** distal portion of female genitalia in detail, lateral (enlarged area marked by rectangle in **D**; closed arrow points to the ovipositor tip; asterisk identifies oviscapt seen by transparency; bracket indicates distal margin of sternum eight). Scale bars: 0.3 mm (**A, B**), 1 mm (**C)**, 0.2 mm (**D, E**).

**Figure 4.**
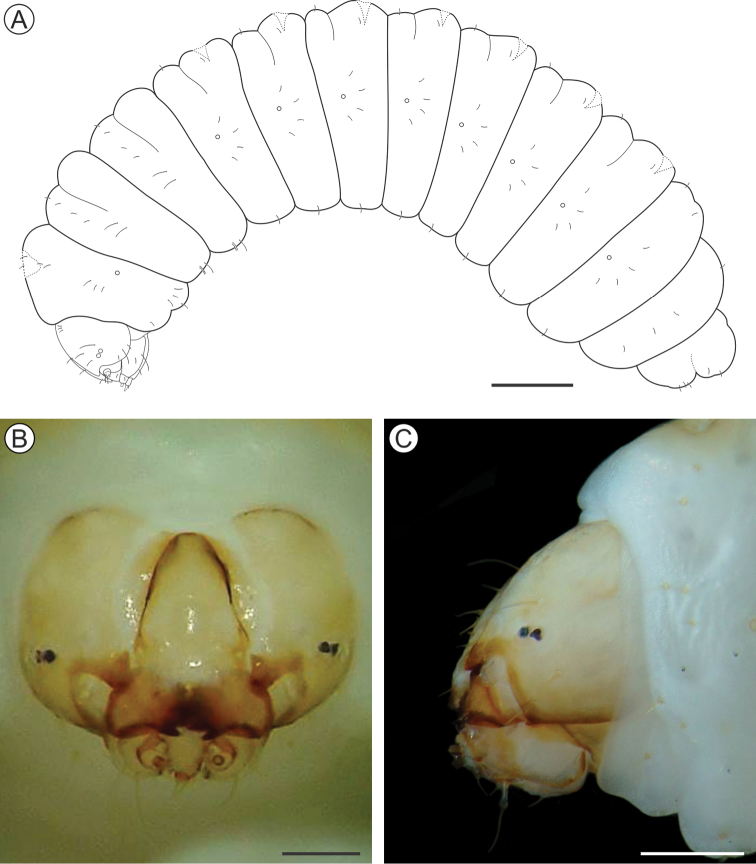
*Andescecidiumparrai* last larval instar under light microscopy (LMCI 218-08). **A** general schematic representation, lateral view **B, C** head, anterior (**B**) and lateral (**C**). Scale bars: 1 mm (**A**), 0.3 mm (**B**), 0.2 mm (**C**).

**Figure 5.**
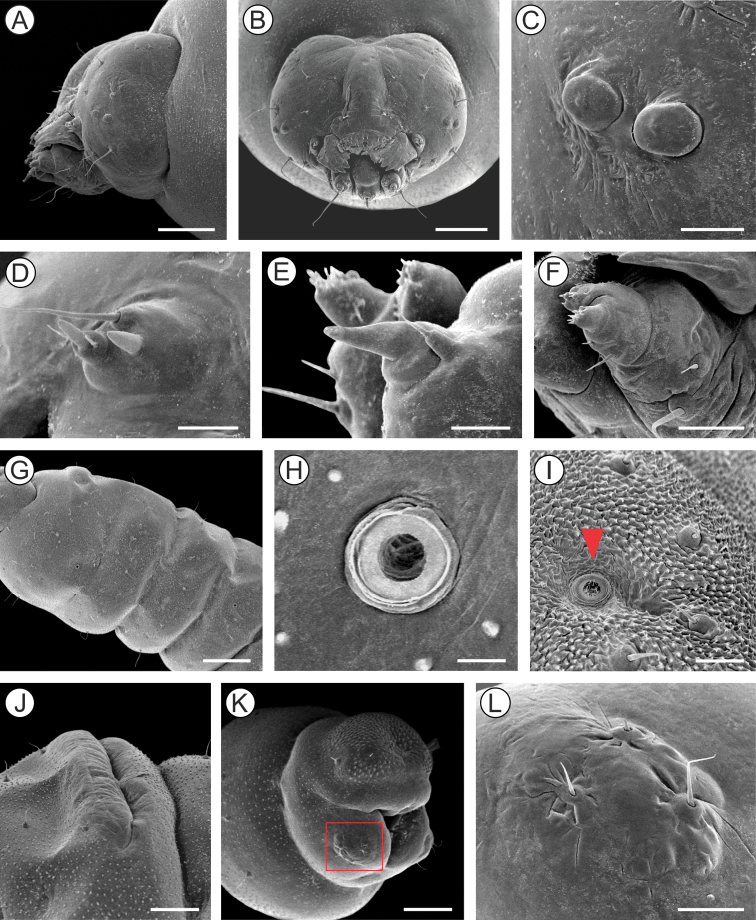
Morphology of *Andescecidiumparrai* last larval instar under scanning electron microscopy. **A, B** head, lateral (**A**) and antero-dorsal (**B**) views **C** stemmata, lateral **D** antenna, lateral **E** labium, lateral **F** maxilla, lateral **G** thorax, lateral **H** first abdominal spiracle, lateral **I** detail of second abdominal segment, showing aligned setae (arrow indicates spiracle), lateral **J** callus of second abdominal tergum, latero-dorsal **K** last abdominal segments, postero-lateral **L** ventral lobe of tenth abdominal segment (enlarged area marked with rectangle in **K**). Scale bars: 150 µm (**A, B**), 30 µm (**C, L**), 20 µm (**D, E**), 50 µm (**F, I**), 300 µm (**G**), 10 µm (**H**), 100 µm (**J, K**).

**Figure 6.**
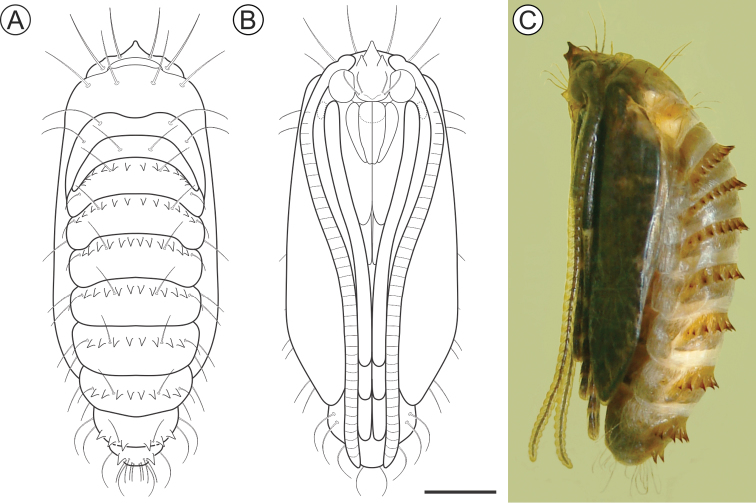
*Andescecidiumparrai* pupa in dorsal (**A**), ventral (**B**), and lateral (**C**) views (LMCI 218-5). Scale bar: 1 mm.

**Figure 7.**
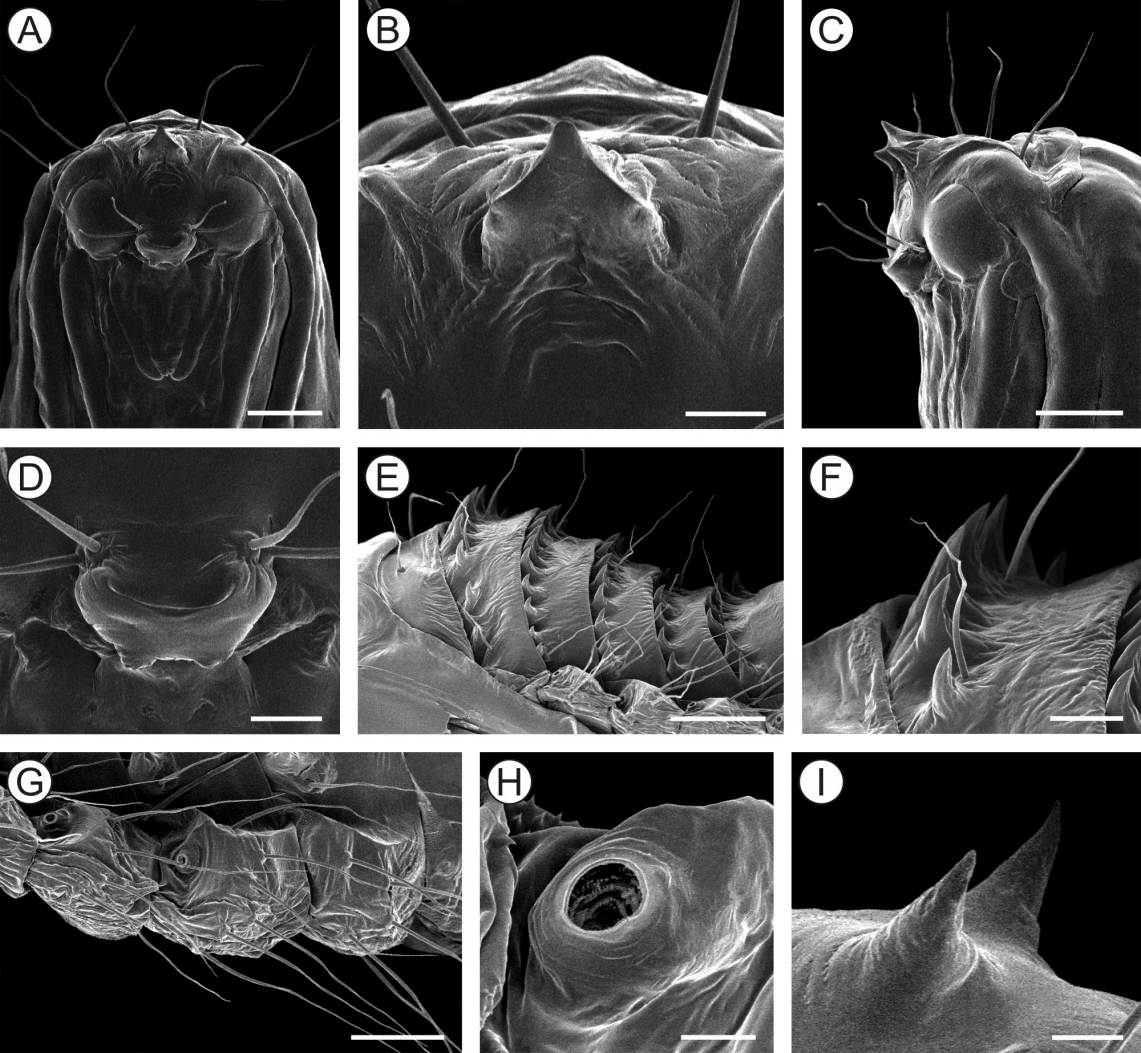
*Andescecidiumparrai* pupal morphology with scanning electron microscopy. **A, C** head and prothorax, under ventral and lateral views, respectively **B** frontal process of head (= gall cutter), ventral **D** clypeus and labrum, ventral **E** abdominal terga, lateral **F** spines and setae of second abdominal tergum in detail, lateral **G** abdominal pleura and sterna, latero-dorsal **H** spiracle of third abdominal segment, dorsal **I** tergal spines of ninth abdominal segment, postero-lateral. Scale bars: 500 µm (**A, C, E**), 150 µm (**B, D, F**), 300 µm (**G**), 30 µm (**H**), 70 µm (**I)**.

**Figure 8.**
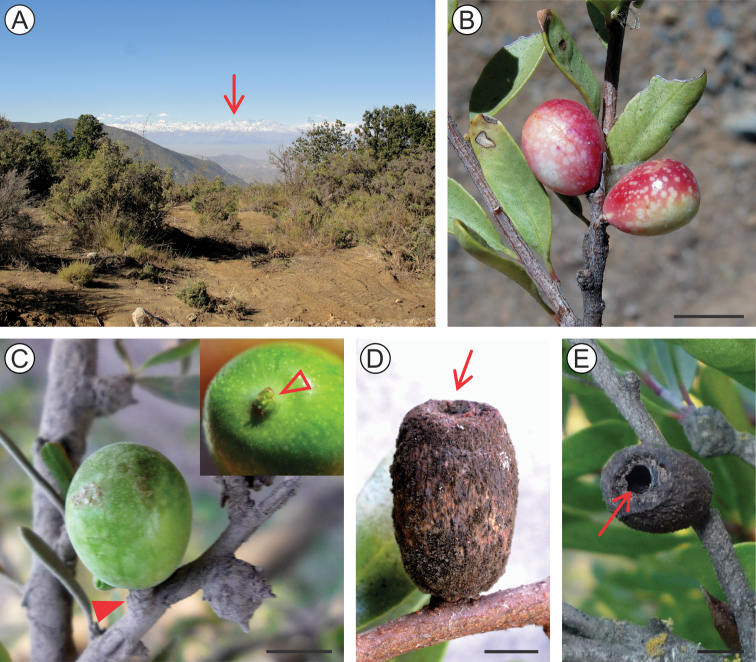
Natural history of *Andescecidiumparrai* on *Schinuspolygamus*. **A** host-plant habitat at Cuesta La Dormida, intersection Til-Til/Valparaíso, beginning of road to Rungue, Chile, 33°3'42"S, 71°0'35"W (seta on the horizon points to the El Altar mountain, Metropolitan region) **B** young, developing galls, lateral **C** mature gall, lateral, with pedunculus (indicated by arrow) in detail in the upper right corner **D, E** old, empty galls under lateral and posterior views, respectively (setae point to apical open). Scale bars: 4 mm (**B, C, E**), 3 mm (**D**).

Figs 2 , 3 , 4 , 5 , 6 , 7 , 8 ; Parra 1998

##### Type species.

***Andescecidiumparrai* Moreira & Vargas, sp. n.**

##### Diagnosis.

*Andescecidium* gen. n. resembles *Cecidonius* in having pupae bearing strong spines on the anterior margins of the abdominal terga and larvae with two stemmata, which make them unique among cecidosids. However, *Andescecidium* shows several adult, larval, and gall features which in conjunction differentiate it from the latter. Adults are much larger, bear mandibular rudiments and have reduced three-segmented maxillary palpi, which are respectively absent and with two in the small *Cecidonius*. The ovipositor in *Andescecidium* is reduced in size, and associated with an inconspicuous dorsal crest in the oviscapt cone that is used to lay eggs on superficial external buds. The ovipositor of *Cecidonius* is very long, having the oviscapt cone dorsally anchored by a conspicuous crest, thus allowing oviposition deep into the stem. Contrary to the larvae of *Cecidonius*, which are unique among cecidosids in having setae of much longer size on the thorax, those of *Andescecidium* show these structures uniform on thorax and abdomen. The spherical galls of *Andescecidium* are associated with stem buds, growing on the external surface from the beginning; they are pedunculate and have fully developed walls. Those of *Cecidonius* grow initially under the bark, erupting through the stem surface later in ontogeny, and with their bases remaining open when mature, clogged with feces.

##### Description of adults

(Figs 2A, B, 3). The adult male morphology was accurately described and illustrated in part by Parra (1998). We complement this description for the male here, adding illustrations that were not provided by Parra, and we also describe the female for the first time.

***Male***. Forewing length ca. 12.1 mm (n = 1). *Head* (Figure 3A): vertex and frons covered by narrow, elongated dark brown scales with a few scattered whitish gray scales. Compound eyes black. Antennae filiform (~ 0.7× length of forewing), with dark brown scales on scape, pedicel and dorsal surface of basal half of flagellum; filiform sensilla on remaining dorsal and ventral surface of flagellum. Labrum semicircular, short. Mandibles poorly developed, as small stubs. Pilifers absent. Maxillae with galeae reduced to small lobes (~2/3 labial palpus length); maxillary palpi tri-segmented (ratios of segments from base ~1.0:0.8:0.8). Labial palpi tri-segmented (ratios of segments from base ~1.0:0.9:1.2). Maxillary and labial palpi with brownish gray scales. *Thorax*. Mostly with brownish gray scales. Anterior arms of latero cervical sclerites not observed. Metafurca (Figure 3B) with slender, elongate postero-dorsal apophyses, free from secondary arms. Forewings (Figure 2A, B) lanceolate, mostly covered with brownish gray scales; a broad dark brown spot close to the middle of the posterior margin of discal cell; a small dark brown spot at apex of discal cell. Hindwings lanceolate, brownish gray. Wing venation as described by Parra (1998: fig. 2). Prothoracic legs (Figure 3C) dark brown with a few scattered whitish gray scales, bearing an epiphysis on distal portion of tibia. Mesothoracic legs similar in coloration to prothoracic legs, with two whitish gray tibial spurs. Metathoracic legs whitish gray with two pairs of tibial spurs; tibia with longitudinal stripe of narrow, elongated hair-like whitish gray scales. Tibial length proportion (anterior / medium / posterior legs) ~ 0.6:0.7:1.0. Abdomen brownish gray with a few dark brown and scattered whitish gray scales. Male genitalia as described by Parra (1998: figs 2, 3, 4A).

***Female***. Forewing length ca. 11.5 mm (n = 2). Similar to male in general, except that sensilla are less abundant and smaller on the antennae, and abdominal sternum VII with caudal margin more sclerotized, bearing a dense ring of stout, elongate setae (Figure 3E). Female genitalia formed by an oviscapt cone (sensu Kristensen 2003, San Blas and Davis 2013), with weak internal dorsal crest, reaching the anterior margin of tergum seven. Anterior apophyses extending to anterior margin of sixth abdominal segment. Posterior apophyses ~1.3× length of anterior apophyses. Posterior apophyses are caudally fused to form the short, compressed, and sagittate apex of the ovipositor. Ductus and corpus bursae membranous, the latter saculiform, without signum.

##### Etymology.

The genus name is derived from a composition between the Portuguese *Andes* and *Cecidia* (a gall; from the Greek *kekídion*). Thus, the epithet refers to the Andes Mountains where the galls of *Andescecidium* were first found.

#### Andescecidium parrai

Taxon classificationAnimaliaLepidopteraCecidosidae

Moreira & Vargas sp. n.

http://zoobank.org/6A158844-5CC2-4F30-AB27-665C2F33E1D8

Figs 2 , 3 , 4 , 5 , 6 , 7 , 8 ; Parra 1998

##### Diagnosis.

As discussed for the monotypic genus.

##### Description of adults.

As discussed for the monotypic genus.

##### Type material.

Chile: Holotype ♂, Cuesta Barriga roadside, Padre Hurtado, Metropolitan Region, emerged June 2013, ex gall on *Schinuspolygamus* collected 22.V. 2013, HA Vargas & GRP Moreira leg. (MNNC). Paratype: 1♀, same data as holotype (MNNC).

##### Non-type material.

Immatures used for morphological description, fixed in Kahle-Dietrich’s fluid, preserved in 70% EtOH: 33°31'24"S, 70°54'35"W, Cuesta Barriga roadside, Padre Hurtado, Metropolitan Region, HA Vargas & GRP Moreira leg., 13 larvae (22.V.2013, LMCI 218-3, 8, 9, 10, 12; 25.XI.2013, LMCI 231-5, 6), 5 pupae (22.V.2013, LMCI 218-3, 5, 11, 14), 3 galls (22.V.2013, LMCI 218-5, 11, 13); 3°3'42"S, 71°0'35"W, roadside on Cuesta La Dormida, border Til-Til/Valparaiso, HA Vargas & GRP Moreira leg., 4 larvae (28.XI.2013, LMCI 233-6), 5 galls (22.V.2013, LMCI 216- 1 to 5, 11); 33°00'31"S, 70°53'52"W, 712m, roadside near Til-Til, Rungue, Metropolitan Region, HA Vargas & GRP Moreira leg., 6 larvae (26.XI.2013, LMCI 232-6, 7), 4 galls (26.XI.2013, LMCI 232-8), G San Blas leg., 12.X.2011, 3 larvae (donated to LMCI 163-14), 46 larvae (IADIZA), 5 galls (IADIZA), 7 pupae (extracted from dried galls on 3.V.2012, IADIZA), G San Blas leg., 15.III.2013, 3 dried pupae; 35°36'00"S, 71°07'17.02"W, 426m, Ruta J-65, 25km from Curicó, Maule Region, G San Blas leg., 12.X.2011, 16 larvae; 36°39'30"S, 72°16'41"W, 43m, roadside in Cruce Nebuco, Bio-Bio Region, HA Vargas, LE Parra & GRP Moreira leg., 2 galls (30.V.2013, LMCI 223-1) ; 35°31'53"S, 71°18'4"W and 35°31'29"S, 71°18'33"W, P. Sta. Edilia, San Clemente, Maule Region, LE Parra & GT Silva leg., 25.XI.2016, 2 larvae donated to LMCI (330-1, 2).

##### Additional larvae.

Same data as above, fixed and preserved in 100% EtOH at -20 °C for DNA extraction: n = 2, LMCI 231-5; n = 6, LMCI 233-5, 6.

##### Additional adult material.

Genitalia preparation on slide of male by LE Parra, UCCC, Parque Nacional La Campana, Sector Ocoa, Hijuelas, Valparaíso Region, L. Sáiz leg., March-April 1993, with label “*Cecidosesargentinana* (Brèthes), Det. LE Parra”; added label “*Andescecidiumparrai* Moreira & Vargas, Det. GRP Moreira; 9.V.2018”, 1♂ (LMCI 218-03-04) and 1♀ (LMCI 218-03-03), cleared in KOH 10%, preserved in 70% EtOH, same data as the holotype.

##### Etymology.

Named in honor of Prof. Dr. Luis E. Parra, from the Universidad of Concepción, who for the first time characterized such a gall-inducing moth, for his great contribution to the development of Lepidopterology in Chile.

##### Description of immature stages.

***Last instar larva*** (Figs 4, 5). Body length = 8.10 ± 0.66 mm; head capsule width = 1.40 ± 0.04 mm (n = 4). *Head* (Figs 4B, C; 5A, B) yellowish with anterior margin light brown; lateral margins convex; frontoclypeus slightly bulged, subtriangular, with well-marked pigmented adfrontal sutures; ecdysial lines unpigmented. Two pairs of laterally located, well-developed stemmata (Figure 5C). Antennae (Figure 5D) 2-segmented; basal segment with one stout and one ~4× longer, filiform sensillum; distal segment with one stout sensillum on apex, flanked by two small filiform sensilla. Labrum slightly bilobed, with five pairs of small setae. Mandible well developed, with four cusps along distal margin (Parra 1998: fig. 4C), bearing one basal seta on external surface. Maxilla with palpus and galea reduced (Figure 5F). Spinneret conical-tubular (Figure 5E; Parra 1998: fig 4D); labial palpus one-segmented with well-developed apical seta. Chaetotaxy consisting of 15 pairs of setae: F group unisetose; C group unisetose; A group trisetose; AF group unisetose; P group trisetose, reduced in length; S group trisetose, middle one long; SS group trisetose, posterior one long.

*Thorax* (T) and abdomen (A) subcylindrical, creamy-white and covered with microtrichia (Figure 5I); thoracic setae small (Figure 5G), similar in size to abdominal ones. Prothoracic shield light yellow, weakly defined; thoracic legs and abdominal prolegs absent; abdominal calli (Figure 5J) on segments A1–A7 located on posterior margin of terga. Last abdominal segment (Figure 5K, L) composed of three lobes, one dorsal, and two ventral, smaller. Spiracles (Figure 5H, I) laterally on T1, A1–A8, circular and without elevated peritreme. Chaetotaxy: T1 with twelve pairs of setae; D group bisetose, SD bisetose, outside prothoracic shield, L group trisetose, anterior to spiracle, SV group trisetose, MV unisetose, V unisetose. T2–3 with nine pairs of setae; D group bisetose, SD bisetose, L group bisetose, SV group bisetose, V unisetose. AB1–8 with seven pairs of setae; D group bisetose, L group tetrasetose, posterior to spiracles, V group unisetose. AB9 with four pairs of setae; D group unisetose, L group bisetose, SV group unisetose. A10 with five pairs of setae; SD group bisetose; SV trisetose.

***Pupa*** (Figs 6, 7). Length = 12.3 + 0.4 mm; n = 3. Yellowish brown, with head, thorax, wings, and abdominal spines becoming dark brown near adult emergence. Head with frontal process (= gall-cutter) formed by three processes (Figure 7A–C); one large, subtriangular, located centrally at the anterior, which is flanked by the other two, shorter, located posteriorly at the base. Antennae narrow, long, slightly surpassing the end of abdomen; prothorax is a narrow transverse band between head and mesothorax; hindwings concealed by forewings, extending to sternum A8; pro-, meso- and metathoracic legs extending up to A6, A7 and A8, respectively. Frons with two pairs of setae laterally. Terga T2 and T3 with two pairs of setae, one dorsal and one latero-dorsal. Abdominal segments with spiracular region covered with microtrichia; A2–9 with a transverse band of conspicuous spines (Figure 7E, F), near anterior margin of terga; tergum A9 with one pair of spines (Figure 7I) on posterior margin. Abdominal setae (Figs 6A, 7G) arranged in three rows (dorsal, supra and subspiracular); one dorsal pair on segments A1–8; one supra-spiracular pair on segments A2–8; four to six subspiracular pairs on segments A2–9; four pairs dorsally on A10; spiracles (Figure 7H) with slightly elevated peritreme, laterally on A2–8.

##### Life history.

The large, short pedunculated galls of *A.parrai* develop externally from the beginning, on stem buds of *S.polygamus* terminal and sub-terminal branches (Figure 8B, C). A few galls of different sizes may be found on the same branch. They are elliptical and usually reddish when young, becoming to spherical and green when mature. The larval chamber is almost cylindrical in shape (maximum width and length varying from 9–10 and from 10–14 mm, respectively; n = 10), and transverse to the stem axis. Their external wall becomes dark brown and almost cylindrical during late development. At least some are apparently deciduous, falling to the ground. This morphotype has a rough external wall (Figure 8D), and was found among the litter under the *S.polygamus* plants, where they are easily confused with fecal pellets of the European rabbit (*Oryctolaguscuniculus* L.: Leporidae), that is abundant in the region. Like *C.pampeanus* galls, those of *A.parrai* also lack an operculum. Using the frontal process and abdominal spines present on the pupae to make pressure, together with body contortions, are likely the means the pupa uses to open an orifice on the distal, weaker wall (Figure 8E), in which it pushes itself partially out of the gall.

Little is known about biology of *A.parrai* galls. Our field observations suggest that the species is univoltine, galls starting to develop during late spring and summer, when most leaf buds are found on *S.polygamus* plants (Sáiz and Núñez 1997). Deciduous galls were field-collected from the ground near the end of May, from which a few adults used here for description successfully emerged in the laboratory after ca. 25 days.

### *Oliera* Brèthes

#### Oliera saizi

Taxon classificationAnimaliaLepidopteraCecidosidae

Moreira & Vargas sp. n.

http://zoobank.org/C959AC54-A09B-4EE5-A35C-2247A3D5744D

**Figure 9.**
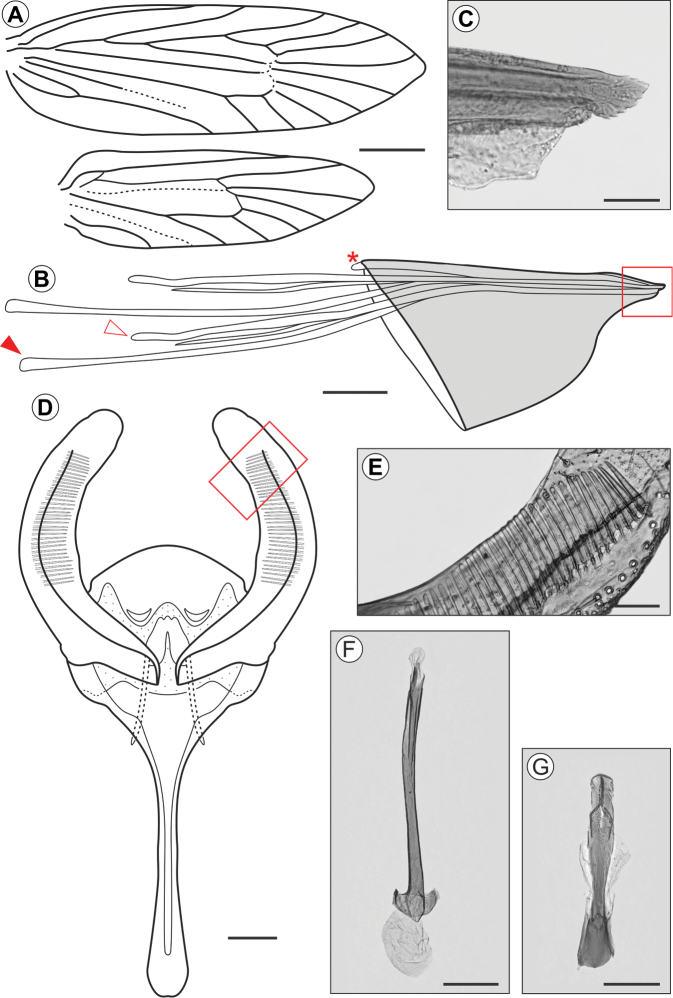
*Olierasaizi* adult morphology, under light microscopy (paratypes). **A** fore and hindwing venation, dorsal view **B** schematic representation of female oviscapt cone, lateral (asterisk indicates the distal end of internal dorsal crest; closed and open arrows point to the anterior ends of anterior and posterior left apophyses, respectively) **C** ovipositor tip in detail, lateral (enlarged area marked by rectangle in **B**) **D** male genitalia, ventral (phallus and juxta omitted) **E** distal section of right valva pectinifer in detail (enlarged area marked by rectangle in **D**) **F** phallus, ventral **G** juxta, ventral. Scale bars: 1 mm (**A**), 200 µm (**B**), 10 µm (**C**), 100 µm (**D, F, G**), 30 µm (**E**).

**Figure 10.**
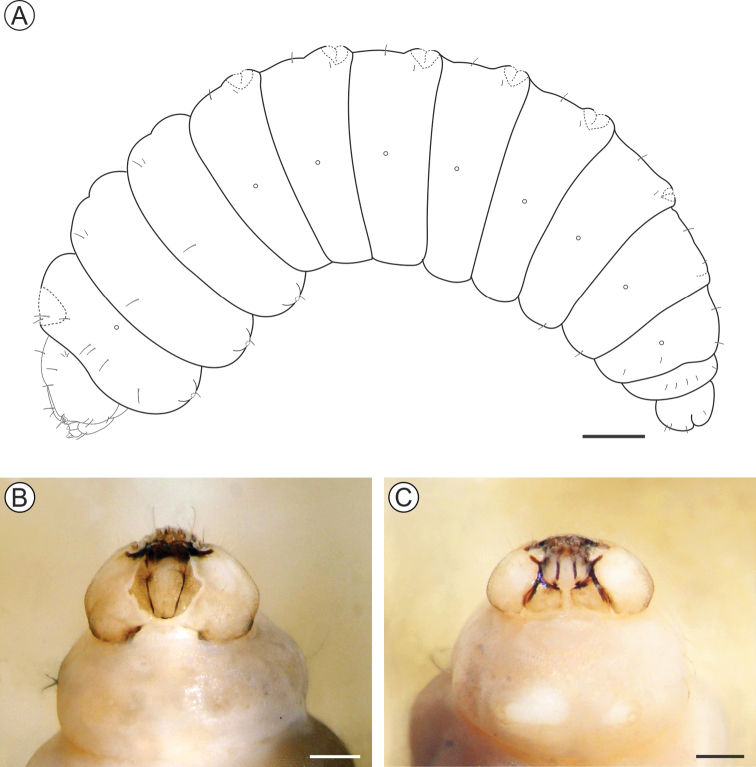
*Olierasaizi* last larval instar under light microscopy (LMCI 231-3). **A** general schematic representation, lateral view **B, C** head and prothorax, dorsal (**B**) and ventral (**C**). Scale bars: 0.5 mm (**A**), 0.3 mm (**B, C**).

**Figure 11.**
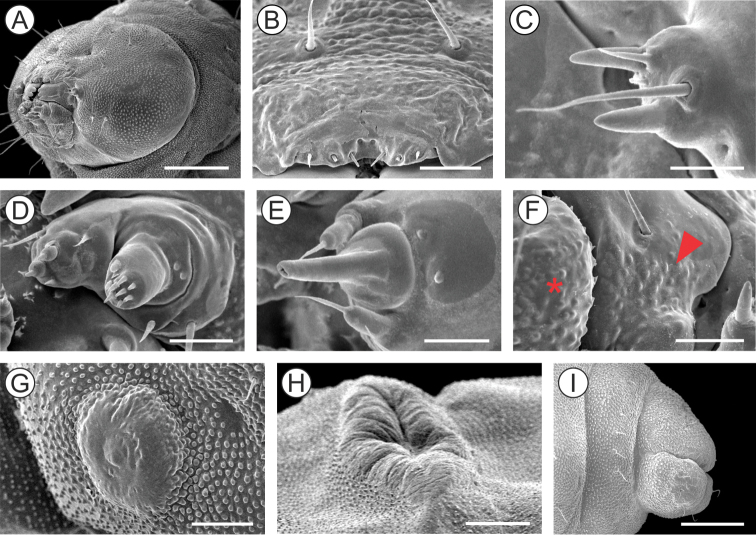
Morphology of *Olierasaizi* last larval instar under scanning electron microscopy. **A** head, antero-lateral view **B** labrum and clypeus, anterior **C** antenna, lateral **D** maxilla, anterior **E** labium, ventral **F** detail of maxilla (indicated by asterisk) and mandibula (pointed by arrow) bases, showing protuberances **G** mesothoracic leg rudiment, lateral **H** callus of third abdominal tergum, latero-dorsal **I** last abdominal segments, lateral. Scale bars: 250 µm (**A**), 40 µm (**B**), 20 µm (**C, E, F**), 30 µm (**D**), 60 µm (**G**), 100 µm **(H**), 200 µm (**I**).

**Figure 12.**
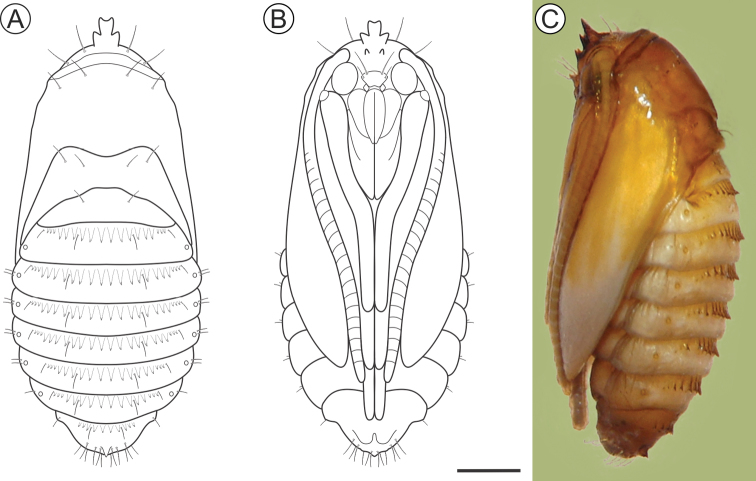
*Olierasaizi* pupa under light microscopy, in dorsal (**A**), ventral (**B**) and lateral (**C**) views (LMCI 233-3). Scale bar: 0.5 mm.

**Figure 13.**
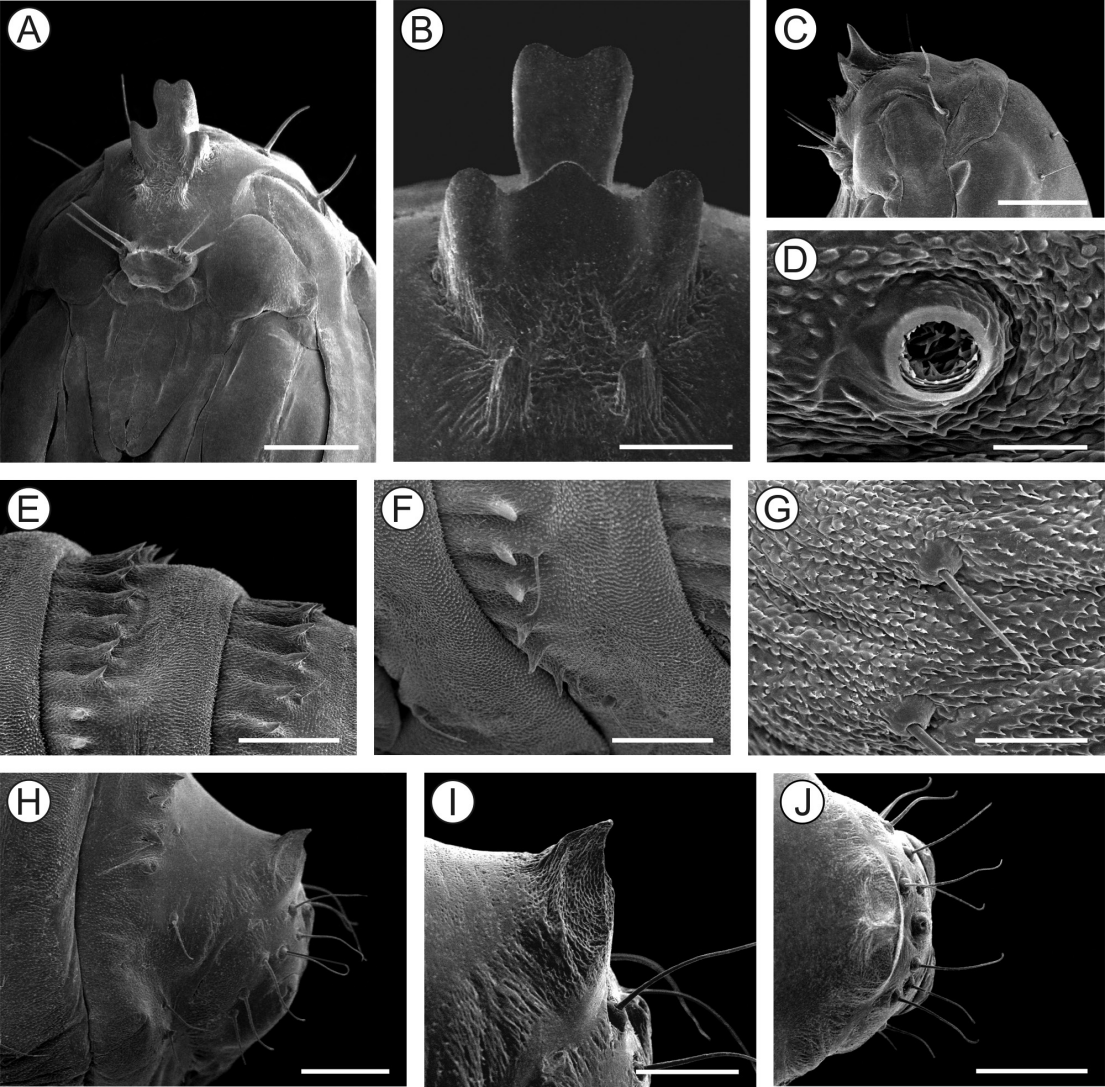
*Olierasaizi* pupal morphology using scanning electron microscopy. **A, C** head and prothorax, under ventral and lateral views, respectively **B** frontal processes of head (= gall cutter), ventral **D** spiracle of second abdominal segment, lateral **E** abdominal terga VI and VII, lateral **F** detail with supraspiracular setae and spines of abdominal tergum VI, lateral **G** detail with subspiracular setae of abdominal segment five, lateral **H, J** last abdominal segments, under lateral and dorsal views, respectively **I** single tergal spine on abdominal segment nine in detail, lateral. Scale bars: 250 µm (**A**), 100 µm (**B, I**), 350 µm (**C**), 30 µm (**D**), 200 µm (**E, H, J**), 150 µm (**F**), 50 µm (**G**).

**Figure 14.**
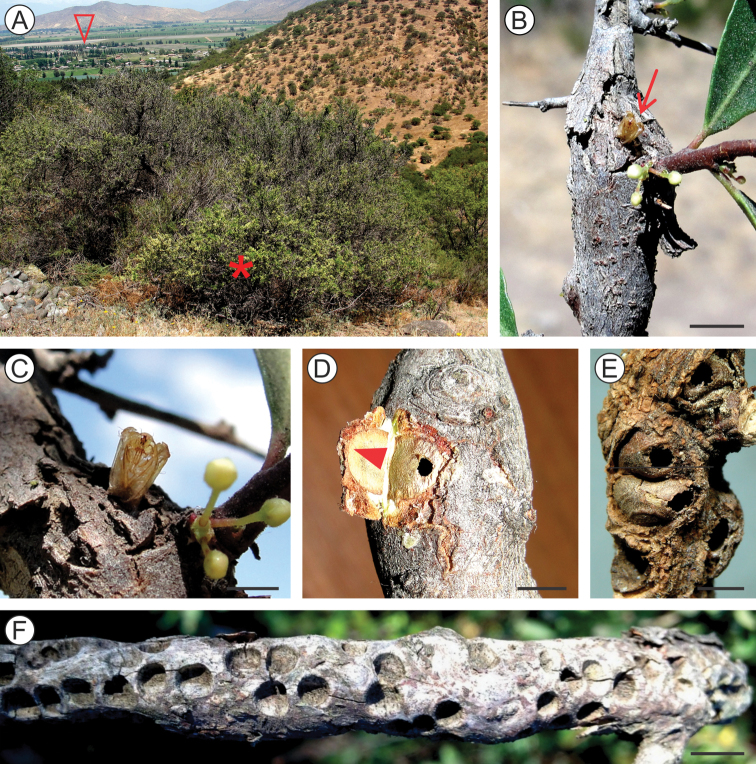
Natural history of *Olierasaizi* on *Schinuspolygamus*. **A** host-plant habitat at Cuesta Barriga, near Santiago city, Chile, 33°31'24"S, 70°54'35"W (asterisk locates the plant; open arrow indicates commune of Padre Hurtado in the valley, Metropolitan region) **B** apical branches showing swollen stem with galls under bark **C** pupal exuvium protruded from the gall exit hole, just after the adult emergence (enlarged area pointed by arrow in (**B**) **D** intact empty gall, shown by detaching the bark (indicated by arrow) **E** empty galls, with decaying gall-wall still remaining, lateral **F** old gall signs appearing as small craters on surface of dried branch. Scale bars: 5 mm (**B**), 2 mm (**C**), 3 mm (**D, E**), 15 mm (**F**).

Figs 2 , 9 , 10 , 11 , 12 , 13 , 14

##### Diagnosis.

A typical member of *Oliera*, having two-segmented labial palpi, pupa with five-pointed frontal process and galls enclosed within swollen terminal branches of *Schinus* plants (for a generic review, see Moreira et al. 2012). It differs from *O.argentinana* by characteristics present on the adults, pupae, larvae, and galls. Contrary to adults of *O.argentinana* that vary from shiny copper to reddish brown, those of *O.saizi* are brownish gray in color. Adults of *O.saizi* (forewing length 5.5–6.3) are slightly larger than *O.argentinana* (forewing length 4.3 mm). Pupae of *O.saizi* have the two lateral units of the anterior row of the gall cutter short, ca. 1/3 the central one in length; in *O.argentinana* such processes are much longer, reaching ca. 3/4 the central in length. Furthermore, pupae of *O.saizi* bear long, conspicuous setae on the last abdominal segment, which are absent in *O.argentinana*. Larvae of *O.saizi* have the head with long, continuous and rectilinear, pigmented adfrontal sutures more or less parallel to the unpigmented ecdysial lines. Together they delimit two long adfrontal areas, which extend from the posterior margin of the labrum to the epicranial notch; in *O.argentinana*, the adfrontal sutures are marked only distally, delimiting two smaller, semicircular, posteriorly located adfrontal areas. In addition, the integument of *O.saizi* is covered by a greater number of rounded microtrichia that are finer and more regular in size compared to the less dense, larger in size and more irregular-shaped ones in *O.argentinana*. Galls of *O.saizi* are larger, and generally found on subterminal branches of *S.polygamus* plants, contrary to those of *O.argentinana* that occur commonly on narrower, terminal branches.

##### Description of adults.

***Male***. Forewing length varying from 5.5 to 6.3 mm (n = 3). *Head*: Vertex and frons covered with smooth, narrow, brownish gray scales. Compound eyes black. Antennae filiform, about half the length of forewing, with brownish gray scales dorsally, ventral half with short sensilla. Labrum semicircular, short. Mandibles poorly developed, as small stubs. Pilifers absent. Maxillae with galeae reduced to small lobes; maxillary palpi tri-segmented (ratios of segments from base ~1.0:0.9:0.6). Labial palpi tri-segmented (ratios of segments from base ~1.0:0.8:0.7). Maxillary and labial palpi with brownish gray scales. *Thorax*: Mostly with brownish gray scales. Wing venation (Figure 9A) as in *O.argentinana* (Moreira et al. 2012). Prothoracic legs with coxa, trochanter and femur dark gray (occasionally brownish gray); tibia and tarsus brownish gray; apex of tibial epiphysis reaching the apex of tibia. Mesothoracic leg brownish gray; one pair of tibial spurs. Metathoracic legs brownish gray; two pairs of tibial spurs; tibia with longitudinal band of narrow, elongated hair-like scales. Tibial length proportion (anterior / medium / posterior legs) ~ 0.5:0.6:1.0. Fore- and hindwings lanceolate, brownish gray. *Abdomen*: Brownish gray. Genitalia (Figure 9D) with narrow tegumen; uncus bilobed; socii as two small setigerous lobes. Saccus with dorsal arms narrow, U-shaped, with anterior projection prominent, slightly widened close to apex. Valva narrow, about 3/4 the length of the anterior projection of the saccus; dorsal and ventral margins sub-parallel, and distal portion dorsally dilated. Pectinifer (Figure 9E) as a straight band on the ventral part of the medial surface of the valva. Transtilla as an inverted “V”, with ventral arms straight, slightly widened close the vertex. Juxta (Figure 9G) narrow, elongated, slightly spatulate distally. Phallus (Figure 9F) narrow, somewhat sinuous, with similar length to valva, with anterior apex laterally widened forming a semicircle; vesica without cornuti.

***Female***. Forewing length varying from 5.2 to 5.9mm (n = 7). Similar to male in general, but with abdominal sternumVII showing a ring of stout setae on caudal margin. Genitalia (Figure 9B) bearing an oviscapt cone (sensu Kristensen 2003, San Blas and Davis 2013), with weak internal dorsal crest, reaching the anterior portion of tergum seven. Posterior apophyses fused posteriorly, forming distally the laterally compressed, serrated apex of the ovipositor (Figure 9C). Anterior apex of posterior apophyses extend to abdominal segment VI; anterior apophyses project into the segment V. Internal reproductive system not represented, apparently as in *O.argentinana* (Moreira et al. 2012).

##### Type material.

Chile: Holotype ♂, Cuesta Barriga roadside, Padre Hurtado, Metropolitan Region, emerged December 2013, ex gall on *Schinuspolygamus* collected 25 November 2013, HA Vargas & GRP Moreira leg. (MNNC). Paratypes: 1♂, 2♀, same data as holotype (MNNC); 7♀, same data as holotype (IDEA); 1♂, roadside near Til-Til, Rungue, Metropolitan Region, emerged December 2013, ex gall on *Schinuspolygamus*, collected 26 November 2013, HA Vargas & GRP Moreira leg. (IDEA).

##### Non-type material.

Immature specimens used for descriptions: 33°31'24"S, 70°54'35"W, Cuesta Barriga roadside, Padre Hurtado, Metropolitan Region, HA Vargas & GRP Moreira leg., 6 larvae (25.XI.2013, LMCI 231-3, 4); 33°3'42"S, 71°0'35"W, roadside on Cuesta La Dormida, near border Til-Til/Valparaiso, Metropolitan Region, HA Vargas & GRP Moreira leg., 8 larvae (20.V.2013, LMCI 216-14), 2 pupae (28.XI.2013, LMCI 233-3); 33°00'31"S, 70°53'52"W, roadside near Til-Til, Rungue, Metropolitan Region, HA Vargas & GRP Moreira leg., 4 larvae (26.XI.2013, LMCI 232-4); 36°48'22"S, 71°44'36"W, roadside near Recinto, Bio-Bio Region, HA Vargas, LE Parra & GR. Moreira leg., 30.V.2013, 12 larvae (LMCI 222-2 to 6) and 2 pupal exuviae (LMCI 222-1).

##### Additional larvae.

Data as above, fixed and preserved in 100% EtOH at -20 °C for DNA extraction (n = 2, LMCI 232-4; n = 2, LMCI 233-2).

##### Additional pinned-dried adults examined.

Puente El Yeso, Cajón del Maipo, Metropolitan Region, R. Charling C. leg., 4 males, with one genitalia (DRD 16536) and one wing (DRD 29925) preparations; 2 females, with one wing and genitalia preparation (DRD 16537), USNM.

##### Etymology.

Named in honor of Prof. Dr. Francisco Sáiz, from the Universidad Católica de Valparaiso, who first noticed the existence of these galls, for his great contribution to the development of Cecidology in Chile.

##### Descriptions of immature stages.

***Last instar larva*** (Figs 10, 11). Body length = 3.99 ± 0.41 mm; head capsule width = 0.97 ± 0.02 mm (n = 3). Head (Figs 10B, C; 11A) creamy to light brown, ~ 2× broader than high, with convex lateral margins. Frontoclypeus triangular, marked by pigmented adfontral sutures extending to apex of epicranial notch that is paralleled by unpigmented ecdysial lines. Adfrontal sutures and ecdysial lines delimit two long adfrontal areas. Hypostomal ridges well marked and divergent; hypostomal lobes trapezoidal. Integument densely covered by rounded microtrichia (Figure 11F). Stemmata absent; antennae (Figure 11C) reduced, with five sensilla, two stout and three filiform; labrum (Figure 11B) slightly bilobed, with three pairs of small setae on distal margin; mandible well developed with four cusps along distal margin and one small seta basally on external surface; maxilla (Figure 11D) with poorly developed palpus and galea; spinneret tubular; labial palpus one-segmented with well-developed apical seta (Figure 11E). Chaetotaxy consisting of 9 pairs of setae: F group unisetose, C group unisetose, AF group unisetose, A group unisetose, L group bisetose, S group trisetose.

Thorax (T) and abdomen (A) creamy white, mostly covered with rounded microtrichia; prothoracic shield weakly defined by a pair of slightly differentiate areas, with reduced microtrichia. A1–7 with well-developed callus (Figure 11H), mesally, close to posterior margin of terga. Thoracic legs (Figure 11G) reduced to circular, unsegmented tubercles; prolegs absent. Spiracles circular, without elevated peritreme, laterally on T1 and A1–8. Abdominal segment 10 composed of three lobes, one dorsal, wider and two ventral (Figure 11I). Chaetotaxy of thorax and abdomen composed of setae reduced in size. T1 with eight pairs of setae; XD group bisetose, SD group unisetose outside prothoracic shield, L group trisetose, SV group unisetose, V group unisetose. T2–3 with four pairs of setae; D group bisetose, L and V groups unisetose. A1–5 with two pairs of setae; D group bisetose. A6–7 with three pairs of setae, D group bisetose and V group unisetose. A8 with four pairs of setae; D group bisetose, L group unisetose and SV group unisetose. A9 with five pairs of setae; SD group unisetose and L group tetrasetose. A10 with four pairs of setae; SD unisetose, SV unisetose and V bisetose.

***Pupa*** (Figs 12, 13). Length = 6.1 + 0.2mm; n = 3. Orange brown, becoming dark brown near adult emergence. Head with frontal process (= gall-cutter; Figure 13A–C) formed by five spines that are grouped into two parallel rows; the anterior row with three processes; middle process with a blunt apex, ca. 3/4 longer than lateral ones; posterior pair consists of two minute, pointed processes. Vertex with two pairs of setae laterally. Antennae narrow, long, with apex slightly surpassing forewing apex. Prothorax as a narrow transverse band between head and mesothorax; forewings reaching sternum A7; hindwings concealed by forewings; metathoracic legs reaching segment A9. Terga T2–3 with two pairs of latero-dorsal setae. Abdominal segments covered with microtrichia; A2–8 with a transverse band of spines (Figure 13E, F) near anterior margin of terga; tergum A9 with acute process (Figure 13H, I) on posterior margin. Setae relatively small, arranged in three rows, from A1 to A8 (dorsal, supra- and subspiracular): one dorsal pair on segments A1–8; one supra-spiracular pair on segments A2–8; two subspiracular pairs on segments A3–7 and one pair on A8. Six pairs of long and stout setaeon last segment (Figure 13J). Spiracles (Figure 13D) circular, without elevated peritreme, laterally on A2–8, and on A8, reduced.

##### Life history.

*Olierasaizi* develops spindle-shaped galls enclosed within swollen stems of *S.polygamus* subterminal branches (Figure 14A, B). The larval chamber is elliptical in shape and transversally located in relation to the stem axis, and as in *O.argentinana*, the gall lacks an operculum. A progressive necrosis extends up the gall wall with the advent of pupation; meanwhile the pupa opens an orifice on the wall with the aid of its frontal process and through body contortions. The abdominal spines help the pupa during these movements to anchor itself on the wall; it pushes part of its body out of the gall, when the exuvia splits and the adult emerges (Figure 14B, D). The outer wall eventually collapses, leaving the empty galls appearing as small holes on the host plant branches (Figure 14E, F).

Little is also known about the biology of *O.saizi*. Similar to what has been found for *O.argentinana* (Moreira et al. 2012), their galls have been collected occasionally, either on isolated plants or in groups. They may occur at high densities per branch, sometimes adjacent to those of *A.parrai*. Our field collection data indicate that the species is univoltine, with adult emergence occurring from late spring to early summer (November / December). *Schinuspolygamus* branches bearing galls with full-grown pupae were field-collected during November, from which a few adults used for description emerged under laboratory conditions ca. 25 days later.

##### Host plant and distribution of both species.

**Figure 15.**
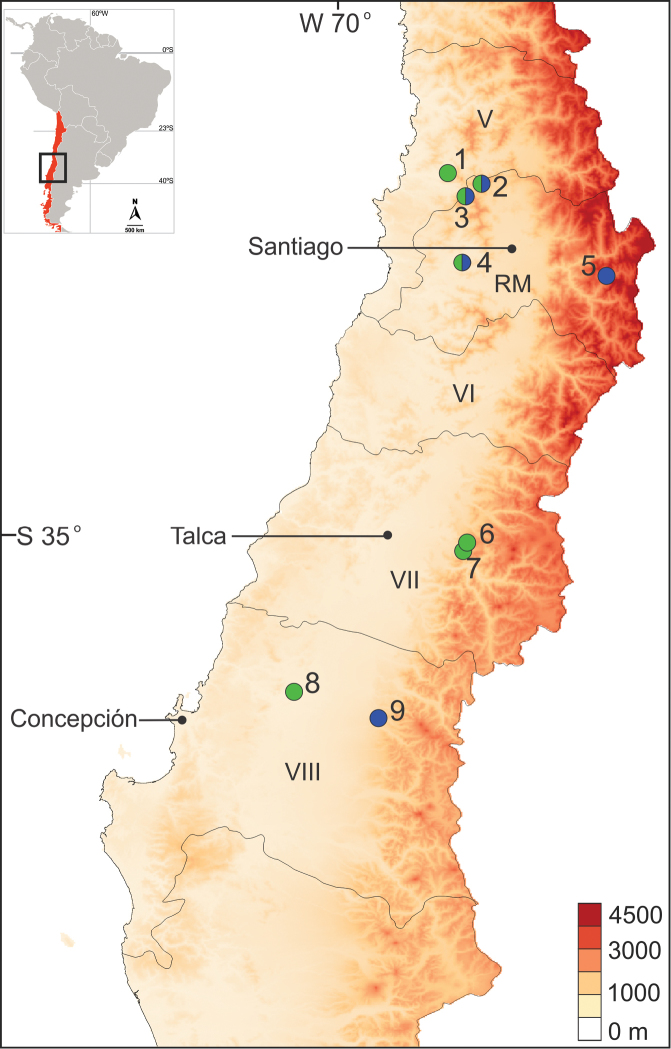
Geographic distribution of *Andescecidiumparrai* (green circles) and *Olierasaizi* (blue circles) in central Chile. Regions are indicated by Roman numbers except for the Metropolitan (RM). Localities are indicated by cardinal numbers: **1** Parque Nacional La Campana **2** Cuesta La Dormida **3** Til-Til/Hungue **4** Cuesta Barriga **5** Cajón del Maipo **6** Curicó **7** San Clemente **8** Cruce Nebuco, and **9** Recinto; for a complete description of corresponding localities, see list of specimens examined.

Galls of both *A.parrai* and *O.saizi* have been found only on branches of *Schinuspolygamus* (Cav.) Cabrera (Anacardiaceae), a small tree with single and glabrous leaves and slender spiny branches (Barkley 1957, Fleig 1989), commonly known in Chile as Huingan. The taxonomy of *Schinus* L. in southern South America is rather complex, as its wide geographic distribution and phenotypic plasticity have lead historically to a confused taxonomy. In fact, the genus is currently under review (CLS Luz, USP, pers. comm.), and *S.polygamus* (sensu lato; Cabrera 1938, Fleig 1987, 1989) may be split into several species, as already proposed by Barkley (1957). Nevertheless, according to this author in this case the true *S.polygamus* would be restricted in distribution to Chile. *Andescecidiumparrai* and *O.saizi* galls were found on populations of *S.polygamus* on the central area, also known as the Mediterranean portion of the country (Quintanilla et al. 2012), which extends from 32°45’N to 37°30’S, abridging the V, VI, VII, VIII and Metropolitan (RM) regions (Figure 15). The main characteristics of this area are the presence of two mountainous ranges, the Andes on the east and the Coastal Range on the west, as well as the central valley located in between them. Plants bearing galls of both cecidosid species were found mainly in the Coastal Range (Figs 8A, 14A). The climate in these valleys is typically austral Mediterranean; the vegetation is autochthonous, composed of sclerophyllous forests, palm communities and coniferous forests and steppe at higher altitudes (Quintanilla et al. 2012, Valencia et al. 2015).

## Discussion

This study illustrates further how important are intensive studies including the immature stages to achieve correct identification of true gall-inducing in Lepidoptera. It confirms preliminary findings of Sáiz and Núñez (1997), which were further supported by Parra (1998), showing that the cylindrical external galls associated with *S.polygamus* in Chile are induced by a cecidosid moth, and not by a beetle as originally suggested by Kieffer and Herbst (1905, 1906) and Houard (1933). In fact, during dissection of these galls we occasionally found an unidentified species of Coleoptera associated with them but only later in ontogeny; small, early developing galls always contained a larva of *Andececidiumparrai*. We believe that this beetle acts as a kleptoparasite (sensu Luz et al. 2015), since apparently it does not change substantially the shape of the galls after usurping them. In addition, we found that galls of *A.parrai* may host other unidentified parasitoids, predators, and successors, thus being complex systems, similar to what has been previously demonstrated for *Cecidoniuspampeanus* (Moreira et al. 2017). Thus we attribute in part the historical confusion involving the taxonomy of cecidosids in Chile to the scarce sampling activity from a taxonomic perspective in the past, and to the fact that galls of *A.parrai* and *O.saizi* are sympatric, occurring sometimes on the same host-plant and branch, as already mentioned. Furthermore, galls of the former are deciduous and those of the latter restricted to under bark, which makes them difficult to detect on plants in the field, and as a consequence also hard to be reared to the adult stage by non-specialists.

*Andescecidiumparrai* and *O.saizi* resulted as unique lineages in the present study from both morphological and molecular analyses. The former appeared as one of the most derived lineages to be evolved within the extant cecidosids. The genetic distance of *A.parrai* is ca. 10% from the nearest related species, an additional undescribed cecidosid existing in Mendoza, Argentina, which was included in the present study for comparison. *Olierasaizi* diverged ca. 6%, from an undescribed *Oliera* species collected at the same latitude in Argentina, and ca. 5% from the type species *O.argentinana*, sampled in southern Brazil. Thus molecular data suggest that although described as monotypic, there is at least one more species belonging to *Andescecidium*, and another to *Oliera*. In fact, these species share morphological characteristics with congenerics regarding the gall and larval stage, but unfortunately they have not been reared yet to the pupal and adult stages, upon which their descriptions are pending.

The present study also provides support to previous statements in the literature in the sense that inclusion of immature stages and galls is essential for elucidation of the taxonomy of Cecidosidae (Moreira et al. 2012, 2017). Our findings indicate that these moths have adults with relatively uniform morphologies, especially in relation to the genitalia, which apparently vary less compared to the immature stages at the generic level (see also Mey 2007). The adult mouth parts, frontal and caudal processes, tergal spines and arrangement of setae on the pupa, number and size of secondary setae on larva, and the plant tissue from which the galls are differentiated in association with their shape and size are apparently the main sources of morphological variation within the group. The wide genetic divergence among congenerics (6% in *Oliera*, 7% in *Cecidonius* and 10% in *Andescecidium*) found up to now at the molecular level (mitochondrial, COI sequences) is also notable, which demonstrates the importance of this kind of analysis in taxonomic studies.

A complete scenario of relationships within Cecidosidae, inferred in the Bayesian calibrated-time tree, retrieved high posterior probabilities for all nodes. It was somewhat unexpected to obtain a fully resolved genus-level tree, based on only two to three markers (see Kawahara et al. 2011). Therefore, results at this level in our study should be interpreted cautiously, as the support provided by the Markov Chain Monte Carlo-based Bayesian method may be overestimated, since it is more dependent on the model suitability than other methods, such as bootstrap (Simmons et al. 2004). The use of more loci usually increases the robustness of phylogenies, particularly the support of nodes (Regier et al. 2009, Kawahara et al. 2011, Zwick et al. 2011). Although COI alone may not accurately reflect the evolutionary relationships among species (Wahlberg and Wheat 2008), it is suitable as a simple estimate of genetic distance among moth lineages, as a first approach to evaluating the amount of difference. Regier et al. (2015) sequenced 15 nuclear genes (ca. 7,500 bp) in distinct Adeloidea families and found 25% of variation within Cecidosidae (*Cecidoses* vs. *Dicranoses*), quite similar to the 23% we found in COI. A comparative analysis using multilocus data generated by Regier et al. (2015) on variation within Adeloidea supports that Cecidosidae present greater variation in relation to the other families included (Adelidae, 13%; Incurvariidae, 13%; Prodoxidae, 17%). The high variation found by Regier et al. (2015) and in our study (also shown by long branches in the reconstructed phylogeny of Cecidosidae) might be related to the old age of this group (i.e. with more time to accumulate differences between lineages), incomplete sampling, and/or early extinctions. Additionally, differences in patterns and/or rates of molecular evolution of cecidosids (also observed in the genus *Scyrotis*, retrieved as polyphyletic) specifically related to COI might be due to metabolism-related factors (Pentinsaari et al. 2016).

In this study we recorded for the first time two new species of cecidosids for Chile that belong to two relatively recent genera (*Andescecidium* and *Oliera*) that apparently have representatives distributed on both the west and east sides of the Andes. Based on a broader survey exploring several southern South America biomes, Silva et al. (2018) suggested that the uplift of the Andes had a major role on speciation of *Eucecidoses* (Brèthes), a related genus with distribution restricted to the eastern Andes. Similar studies should be conducted for the west Andean cecidosid species described here. As already mentioned, galls induced by *O.saizi* had not been detected on *S.polygamus* before this study. But gall morphotypes similar to them, and also to those described in this study for *A.parrai*, were reported for the stems of *S.latifolius* (Gillies ex Lindl.) Engl. by Sáiz and Núñez (1997). The geographic distribution of *S.polygamus* is broader than that covered in this study, and there are a few other native species of *Schinus* in Chile (see Rodríguez et al. 2018), which should be also explored for galls. In other words, the diversity of cecidosids in Chile is believed to be greater, and thus should be better studied from a taxonomic perspective, involving other Anacardiaceae species, before a phylogeographic approach can be undertaken.

## Supplementary Material

XML Treatment for Andescecidium

XML Treatment for Andescecidium parrai

XML Treatment for Oliera saizi
